# Supplementary material for: Incorporating usability evaluation into iterative development of an online platform to support research participation in Parkinson’s disease: a mixed methods protocol
Source: BMJ Open. 2023 Dec 19;13(12):e078638. doi: 10.1136/bmjopen-2023-078638 (PMC10748888; doi:10.1136/bmjopen-2023-078638)
Supplement: Supplementary data [file bmjopen-2023-078638supp001.pdf]

People with Parkinson's and care partners) Interview Registration Form [to be inserted into JISC survey)

Section 1
The following data is collected so that we are able to contact you to arrange an interview:

First Name:
Surname:
Date of Birth:
Email Address:
Phone Number:

Have you been using the website as a person with Parkinson's or as a care partner?

- Person with Parkinson's
- Care partner

Preferred method of contact:

- Email
- Phone

Section 2
We would like to ensure that the website is as user friendly as possible for everyone. By telling us a little bit more about yourself, you will help us achieve this goal.

Post Code:

Gender
Please select your gender

- Male
- Female
- Other (please specify)

Ethnicity
Please select your ethnicity:

- Asian/Asian British – Indian
- Asian/Asian British – Pakistani
- Asian/Asian British – Bangladeshi
- Asian/Asian British – Chinese
- Asian/Asian British – Any other Asian Background
- Black/African/Caribbean Background – African
- Black/African/Caribbean Background – Caribbean
- Black/African/Caribbean Background – Any other Black/African/Caribbean background
- Mixed Ethnic Group – White and Black Caribbean
- Mixed Ethnic Group – White and Black African

- Mixed Ethnic Group – White and Asian
- Mixed Ethnic Group – Any other mixed ethnic group
- Other Ethnic Group – Arab
- Other Ethnic Group – Berber Arab
- Other Ethnic Group – Ashkenazi Jewish
- White – English/Welsh/Scottish/Northern Irish/British
- White – Irish
- White – Gypsy or Irish Traveller
- White – Any other background

Parkinson's disease diagnosis (not applicable for care partners)

- When were you diagnosed with Parkinson's disease? (please give an approximate date if you are not sure) (dd/mm/yyyy)

Familiarity with digital technology

Please indicate if you are able to do any of the following tasks on a computer, phone or tablet

<u>Managing information</u>

- Use a search engine to look for information online
- Find a website I have visited before
- Download/save a photo I found online

<u>Communicating</u>

- Send a personal message via email or online messaging service
- Carefully make comments and share information online

<u>Transacting</u>

- Buy items or services from a website
- Buy and install apps on a device

<u>Problem solving</u>

- Verify sources of information I found online
- Solve a problem with a device/digital service using online help

<u>Creating</u>

- Complete online application forms which include personal details
- Create something new from existing online images, music or video
