# Supplementary material for: Incorporating usability evaluation into iterative development of an online platform to support research participation in Parkinson’s disease: a mixed methods protocol
Source: BMJ Open. 2023 Dec 19;13(12):e078638. doi: 10.1136/bmjopen-2023-078638 (PMC10748888; doi:10.1136/bmjopen-2023-078638)
Supplement: Supplementary data [file bmjopen-2023-078638supp002.pdf]

Researcher Interview Registration Form (to be inserted into JISC survey)

Section 1

The following data is collected so that we are able to contact you to arrange an interview:

First name:
Surname:
Date of birth:
Phone number:
Email address:
Site name:
Site Post Code:
Study name:
Preferred method of contact:

- Email
- Phone

Section 2

We would like to ensure that the website is as user friendly as possible for everyone. By telling us a little bit more about yourself, you will help us achieve this goal.

Please tell us how you have been using the website

- As a researcher
- As a site coordinator
- As both a researcher and site coordinator

Gender
Please select your gender

- Male
- Female
- Other (please specify)

Ethnicity
Please select your ethnicity:

- Asian/Asian British – Indian
- Asian/Asian British – Pakistani
- Asian/Asian British – Bangladeshi
- Asian/Asian British – Chinese
- Asian/Asian British – Any other Asian Background
- Black/African/Caribbean Background – African
- Black/African/Caribbean Background – Caribbean
- Black/African/Caribbean Background – Any other Black/African/Caribbean background
- Mixed Ethnic Group – White and Black Caribbean
- Mixed Ethnic Group – White and Black African

- Mixed Ethnic Group – White and Asian
- Mixed Ethnic Group – Any other mixed ethnic group
- Other Ethnic Group – Arab
- Other Ethnic Group – Berber Arab
- Other Ethnic Group – Ashkenazi Jewish
- White – English/Welsh/Scottish/Northern Irish/British
- White – Irish
- White – Gypsy or Irish Traveller
- White – Any other background

What type of Trust is your site a part of?
Please select the most relevant Trust type

- Large Acute
- Medium Acute
- Small Acute
- Mental Health
- Other (please specify)
- Not known

How many Parkinson's disease studies have you coordinated?

- Please specify the number of Parkinson's disease studies you have coordinated at your current site, and any previous sites you have worked at. If you are not sure, please give a rough estimate.

Familiarity with digital technology

Please indicate if you are able to do any of the following tasks on a computer, phone or tablet

<u>Managing information</u>

- Use a search engine to look for information online
- Find a website I have visited before
- Download/save a photo I found online

<u>Communicating</u>

- Send a personal message via email or online messaging service
- Carefully make comments and share information online

<u>Transacting</u>

- Buy items or services from a website
- Buy and install apps on a device

Problem solving

- Verify sources of information I found online
- Solve a problem with a device/digital service using online help

Creating

- Complete online application forms which include personal details
- Create something new from existing online images, music or video
