# Supplementary material for: Incorporating usability evaluation into iterative development of an online platform to support research participation in Parkinson’s disease: a mixed methods protocol
Source: BMJ Open. 2023 Dec 19;13(12):e078638. doi: 10.1136/bmjopen-2023-078638 (PMC10748888; doi:10.1136/bmjopen-2023-078638)
Supplement: Supplementary data [file bmjopen-2023-078638supp004.pdf]

Prototype Topic Guide for Interviews with Researchers

Interviewer introduces themselves

Before we start, is it okay with you if I audio-record this session?

- The reason we record is so we can go back and remember what was said and what wasn't said. Destroyed as soon as study is done, any publications don't use names
    - *Ask again for confirmation once recording is on*
    - If no: "That's fine, is it okay if I type notes as we talk and share those notes with you once we have finished the interview so you can make sure they are accurate?"

The purpose of this study is find out about your experiences of using the website, so we can find out what is working well or what needs to be changed or improved. Your feedback is very valuable for us to evaluate the website.

Today we're going to be going through some questions to discuss your experiences using the website, and then you will have the opportunity to provide feedback on anything else you want to talk about that you feel we haven't covered. If there is anything you don't understand during the interview, feel free to ask!

Before we get into those questions, I just wanted to remind you that all of your answers will be kept confidential and stored on a password-protected computer that only the research team can access. The audio recording that we're taking today will be deleted as soon as it has been transcribed, and any identifying information (like your name) will be removed from the audio recording before it is sent to the transcription service.

So before we get started, do you have any questions?

1. **General usability**

- How would you describe your experience of using the website?
    - *How much did you like or dislike using the website?(Why was this? What made you like/dislike that aspect?)*
    - *How easy or difficult did you find the website to use? (Why was this? What made that aspect easy or difficult to use?)*

2. **Registration**

- How did you find the process of registering for the website?
    - Do you feel there is anything that could be improved in terms of being registered as a site study coordinator or researcher for your study? If so, what?

3. **Inviting and enrolling participants**

- Did any volunteers send a request to participate in your study?
    - Have they been enrolled onto the study?
    - If no, why not?
- Did you invite any volunteers to enrol on your study?

  - If no, why not?
- Once a volunteer had requested participation, or accepted the study invite, what did you find easy about the enrolment process?
  - What made it tricky?
  - Could anything have been clearer?
  - Did you feel there was anything else that could be improved? If so, what?

4. General feedback

- What would you suggest to make the website better/easier to use/easier to navigate/functions that you would like to see?
  - *Can you tell me more about that, and how you think it would improve the website?*
  - *Are there any other suggestion that you think would make the website better?*
- Is there anything else you would like to mention?
