# Supplementary material for: Incorporating usability evaluation into iterative development of an online platform to support research participation in Parkinson’s disease: a mixed methods protocol
Source: BMJ Open. 2023 Dec 19;13(12):e078638. doi: 10.1136/bmjopen-2023-078638 (PMC10748888; doi:10.1136/bmjopen-2023-078638)
Supplement: Author's manuscript [file bmjopen-2023-078638.draft_revisions.pdf]

BMJ Open is committed to open peer review. As part of this commitment we make the peer review history of every article we publish publicly available.

When an article is published we post the peer reviewers' comments and the authors' responses online. We also post the versions of the paper that were used during peer review. These are the versions that the peer review comments apply to.

The versions of the paper that follow are the versions that were submitted during the peer review process. They are not the versions of record or the final published versions. They should not be cited or distributed as the published version of this manuscript.

BMJ Open is an open access journal and the full, final, typeset and author-corrected version of record of the manuscript is available on our site with no access controls, subscription charges or pay-per-view fees (http://bmjopen.bmj.com).

If you have any questions on BMJ Open's open peer review process please email info.bmjopen@bmj.com

BMJ Open

Incorporating usability evaluation into iterative development of an online platform to support research participation in Parkinson's disease

Journal:	*BMJ Open*
Manuscript ID	bmjopen-2023-078638
Article Type:	Protocol
Date Submitted by the Author:	07-Aug-2023
Complete List of Authors:	Chapman, Rebecca; University of Plymouth, Applied Parkinson's Research Group, Faculty of Health<br>Zeissler, Marie-Louise; University of Plymouth, Applied Parkinson's Research Group, Faculty of Health<br>Meinert, Edward; University of Plymouth, Centre for Health Technology, Faculty of Health ; Newcastle University, Translational and Clinical Research Institute<br>Mullin, Stephen; University of Plymouth, Applied Parkinson's Research Group, Faculty of Health<br>Whipps, Sue; University of Plymouth, Patient and Public Involvement (PPI) representative<br>Whipps, John; University of Plymouth, Patient and Public Involvement (PPI) representative<br>Hockey, Kate; University of Plymouth, Patient and Public Involvement (PPI) representative<br>Hockey, Philip; University of Plymouth, Patient and Public Involvement (PPI) representative<br>Carroll, Camille; University of Plymouth, Applied Parkinson's Research Group, Faculty of Medicine and Dentistry
Keywords:	Parkinson-s disease < NEUROLOGY, QUALITATIVE RESEARCH, REGISTRIES, Surveys and Questionnaires

SCHOLARONE™
Manuscripts

Incorporating usability evaluation into iterative development of an online platform to support research participation in Parkinson's disease

Authors: Rebecca Chapman[1], Marie-Louise Zeissler[1], Edward Meinert[2, 3, 4], Stephen Mullin[1], Sue Whipps[5], John Whipps[5], Kate Hockey[5], Phil Hockey[5], Camille Carroll[1, 4]

Affiliations:

[1] Applied Parkinson's Research Group, University of Plymouth, Faculty of Health, Plymouth, United Kingdom; [2]Centre for Health Technology, University of Plymouth, Faculty of Health, Plymouth, United Kingdom; [3]Primary Care and Public Health, Imperial College London, London, United Kingdom; [4]Translational and Clinical Research Institute, Newcastle University, Newcastle, UK;

[5]Patient and Public Involvement Representative

For peer review only

ABSTRACT

Introduction

Many people with Parkinson's (PwP) are not given the opportunity or do not have adequate access to participate in clinical research. To address this, we have co-developed with users an online platform that connects PwP to clinical studies in their local area. It enables site staff to communicate with potential participants and aims to increase the participation of the Parkinson's community in research. This protocol outlines the mixed methods study protocol for the usability testing of the platform.

Methods and Analysis

We will seek user input to finalise the platform's design, which will then be deployed in a limited launch for beta testing. The beta version will be used as a recruitment tool for up to 3 studies with multiple UK sites. Usability data will be collected from the two intended user groups: PwP or care partners acting on their behalf and site study coordinators. Usability questionnaires and website analytics will be used to capture user experience quantitatively, and a purposive sample of users will be invited to provide further feedback via semi-structured interviews. Quantitative data will be analysed using descriptive statistics, and a thematic analysis undertaken for interview data. Data from this study will inform future platform iterations.

Ethics and Dissemination

Ethical approval was obtained from the University of Plymouth (3291; 3rd May 2022). We will share our findings via a 'Latest News' section within the platform, presentations, conference meetings and national PwP networks.

Strengths and Limitations of this Project

- A mixed methods approach will utilise both qualitative and quantitative methods and enhance our understanding of any usability issues identified in the development of the platform
- We will seek feedback on usability from recruiting staff at study sites as well as patients
- Purposive sampling for semi-structured interviews will ensure inclusivity in terms of demographics, geographical location, and digital literacy, to ensure issues are identified from a broad range of users
- Platform users may not have utilised the whole website prior to interview, and so their answers may not capture the entirety of the platform

INTRODUCTION

Delays in reaching recruitment targets represent a major challenge for clinical trials (1). A reduction in in-person clinic attendance with the introduction of new, remote care delivery models following the COVID 19 pandemic has further exacerbated this problem (2). Parkinson's disease (PD) trials generally do not recruit representative populations and therefore their results are not generalisable, which risks perpetuating healthcare inequalities (3). Strategies to improve recruitment to trials have been evaluated, but other than telephone reminders and opt-out strategies, very few have been found to be effective (4). The creation of an online recruitment tool to facilitate communication with and recruitment of research-interested PD patients has the potential to increase the efficiency of recruitment to PD studies.

For peer review only - http://bmjopen.bmj.com/site/about/guidelines.xhtml

In 2007 we developed a paper-based register of research-interested PD patients within the South West of England. The Parkinson's Register of the Dementias and Neurodegenerative Diseases Research Network (PRO-DeNDRoN) has been previously evaluated, and recognised as a successful and resource efficient recruitment tool, with 85% of registered PRO-DeNDRoN recruiters reporting that the register was a useful means of facilitating research and providing data for planning of service provision (5). However, this required manual data entry resulting in administrative burden, and highlighted the need for a more efficient, online recruitment platform that is easily accessible to people with Parkinson's and time efficient for trial delivery staff.

Online platforms have been used in other disease areas as a successful means of trial recruitment. For example, 'Join Dementia Research' (JDR) is a website that connects people with dementia to research projects across the UK (6). The website has been established in 2015 and in December 2021 JDR reported to have a population of nearly 50,000 research-interested people registered, 12% of which had a self-declared diagnosis of dementia, with 51% of these being women (7). This highlights the potential of online registers as a useful tool for disease-specific recruitment.

Originating from the PRO-DeNDRoN register, we have developed an online platform, conceptualised and designed with PwP and care partner input (see Figure 1 for project milestone overview). Our platform aims to connect people with Parkinson's (PwP) with research projects in their preferred geographical locations. The platform matches participants' eligibility and preferences to open studies and also enables site coordinators to communicate directly with potential participants. The platform has been designed with multi-account permissions in place: central study coordinators (known as 'researchers') who can request to upload a study, administrative staff who review the study documentation, PwP wishing to engage with research and care partners wishing to register PwP for studies on their behalf. Care partners in this context are unpaid and are defined as "the primary person who feels responsible for, and supports, the PwP". There is also an account for study coordinators at each individual study site (created automatically once a study has been approved), who can engage with interested or eligible participants (see Figure 2 for an overview of website functionality).

This project aims to evaluate platform usability and accessibility in the two main user groups, to understand barriers and facilitators to engagement and use.

METHODS

Project design

We will invite usability feedback from PwP, care partner and site coordinator representatives to identify any major bugs or functionality issues in the platform prior to beta testing. The platform will then be deployed in a limited launch to enable mixed-methods evaluation via validated usability questionnaire, website analytics and semi-structured interviews.

Dissemination of platform beta-version

Prior to deployment, we will select up to 3 multi-centre studies in Parkinson's disease actively recruiting within the UK, which have good geographical spread of sites. We will invite research investigator teams to input their study details onto the platform. The beta-version of the platform will then be disseminated to the Parkinson's community via national patient and carer networks, as

For peer review only - http://bmjopen.bmj.com/site/about/guidelines.xhtml

well as charity stakeholders which will include Steering Group meetings and advertisements through research information emails.

Study recruitment and participant selection

Usability questionnaires

All platform users will be invited to complete an online questionnaire which will be available to complete at any time. This will be visible as a tab on the user account menu (for PwP and care partners) or an automated email following account activation (for site coordinators) where users can sign up separately for the questionnaire and semi-structured interviews. A link will divert users to Joint Information Systems Committee (JISC) online surveys, displaying an information sheet and e-consent form. A survey will then capture user type, ethnicity, age and socioeconomic status, followed by the usability questionnaire.

Website Analytics

Upon first visiting the platform, a pop-up will be displayed to ask if the user consents to the use of analytical website cookies. If the user consents, measurement of key performance indicators (KPIs) will be captured.

Semi-structured interviews

All users will be invited to register interest in participating in remotely conducted interviews to gain an understanding of how they experienced aspects of their user journey, as well as whether and how user experience could be improved. A separate question will be displayed on the same tab as for the usability questionnaires or automated email. For each group, the question will ask if they are interested in providing feedback via an interview and contain two links: one to the information sheet and one to the e-consent form. The information sheet will detail the purpose and nature of the interviews, information on what they involve, details regarding free choice and right to withdraw and will describe the retention of data provisions. The information sheet will be accessed via separate link to the consent form to allow users adequate time to consider the nature of the study and ask any questions they have. Once e-consent has been obtained, a JISC demographic survey will be displayed to facilitate purposive sampling (please see Supplementary Files 1 and 2 for a list of demographic questions).

Participants will comprise two intended platform user groups: PwP/their care partners and researchers/site study coordinators and selected purposively. PwP/care partners will be selected based on ethnicity, socioeconomic status, disease duration (if applicable) and digital literacy. Researchers and site coordinators will be selected based on ethnicity, site type, PD research experience and digital literacy. If selected for interview, the participant will be contacted by a member of the project team via their preferred method indicated on the registration survey and arrange a suitable time to conduct the interview. If not selected, the user will receive an email explaining this and thanking them for their time.

Inclusion criteria for all data collection methods

PwP/care partners

- Age ≥18 years
- Diagnosis of Parkinson's disease/ care partner of someone with PD (care partners in this context are unpaid and defined as "the primary person who feels responsible for, and supports, the PwP")
- Prior experience of using a computing device (including but not limited to PC, Mac & Tablet)

For peer review only

For peer review only - http://bmjopen.bmj.com/site/about/guidelines.xhtml

- Has access to a desktop or laptop computer with internet connection
- Willing and able to give informed consent for participation in the project
- Willing and able to comply with project requirements

Researchers/Site Study Coordinators

- Age ≥18 years
- Registered as a named central study coordinator and/or site study coordinator for a study registered on the platform
- Prior computer experience (PC or Mac)
- Willing and able to give informed consent for participation in the project
- Willing and able to comply with project requirements

The only specified exclusion criteria for all website users are being unable or unwilling to provide informed consent or comply with project requirements.

Data Collection

Demographic Information

Demographic data will be collected for all users who agree to complete the usability questionnaire and/or semi-structured interviews. Postcode will be collected to allow for capture of both rural and urban participants, as well as calculation of socioeconomic status via the Index of Multiple Deprivation (IMD) calculator (8). This tool provides the official measures of relative deprivation for small areas in England, and the equivalent tools will be used for the devolved nations (9-11). Socioeconomic status will be captured via the IMD calculator and digital literacy will be captured by reproducing the Lloyds Bank Basic Digital Skills Measure (2018) (12). This is a list of 11 digital tasks split over five skills categories. Respondents are asked which ones they would be able to complete if asked and are classified as having full basic digital literacy skills if they can complete at least one task in each category.

Usability Questionnaires

Questionnaire feedback will be captured using the Telehealth Usability Questionnaire (TUQ) (13), which is suitable for the collection of opinions from both platform user groups (patients and clinical study site staff). It evaluates the usability of telehealth services and is based on six criteria including usefulness, ease of use and learnability, interface quality, interaction quality, reliability and satisfaction and future use. All questions are optional which allow the measure to the tailored to meet the requirements of specific digital services.

Semi-structured interviews

Semi-structured qualitative interviews will be conducted by a University of Plymouth researcher and take place either over the phone or via teleconferencing software and will last no longer than one hour. The participant will be facilitated through the interview process, and prompts will be used to enable guided conversations using an interview guide (see Supplementary Files 3 and 4 for prototype interview questions). This guide has been informed by the MOLD-US framework (14), which allows for results to be classified and interpreted based on impediments that are intrinsic to usability issues experienced by older adults and will also be informed by the initial usability feedback prior to live deployment. The guide contains questions that seek feedback on each aspect of website functionality, from user registration to confirmation of study eligibility (if applicable) and participants will be given the opportunity to identify what went well or what could be improved. All interview

For peer review only - http://bmjopen.bmj.com/site/about/guidelines.xhtml

sessions will be recorded via video and/or audio capture and will be fully transcribed by a University of Plymouth researcher.

Website analytics

Key performance indicators (KPIs) of user behaviour will be measured using HotJar analytical software (15), where users consent for this to be captured through the acceptance of analytical website cookies. General user behaviour will be captured through the use of heatmaps, and individual user journeys will be mapped and navigation timings recorded.

Further performance indicators will include the number of PwP registered to the platform, the percentage requesting and/or invited to take part in a study, and the percentage of PwP accepted for study screening and enrolment.

Sample Size

TUQ

We determined a 95% confidence interval, an accuracy of +/-5 percentage units and a satisfaction with the website random estimation of 50%. Taking into account a target population limit of 500 participants that can be supported within the test-server the sample size needed is 218 participants (16).

Web analytics

We will gather data from all users who consent to the use of website analytics, but we will aim for 10 per user group, per data capture method.

Semi-structured interviews

A purposive sample of 20 users will be selected for semi-structured interviews (10 per participant group) will be selected. While still allowing for a richly textured understanding of the usability issues (17), it is maintained that little new information is generated after completing 20 qualitative interviews.

Data analysis

Quantitative data

Median (range) data will be collated on all usability questionnaire items as an indicator of usability levels for both user groups. For website analytics, average timings to complete user journey subtasks and individual recorded user journeys of "slow" and "fast" completers will be descriptively analysed to gain insights into processes that may cause difficulties. Feedback specific analysis will also be undertaken and depending on interview feedback, particular parts of website user journeys may be descriptively analysed further to gain insights into how optimal improvements can be made.

Qualitative data

For initial feedback prior to beta-testing, we will evaluate the risk of harm according to standard processes (18) which comprise an evaluation of task criticality, frequency and impact. Ratings with high severity of harm ratings will be prioritised for amendment over issues with less high ratings.

For peer review only - http://bmjopen.bmj.com/site/about/guidelines.xhtml

Interview data will be stored, managed and analysed with NVivo. Thematic analysis will be undertaken using the six-step approach of Braun and Clarke: 1) become familiar with data, 2) generate initial codes, 3) search for themes, 4) review themes, 5) define themes, 6) write-up (19).

Project Management

A project management team consisting of researchers, clinicians and PPI representatives will meet monthly and take on the role of data monitoring and project conduct.

All project data will be managed in line with local and national GDPR requirements. All digital data (including digital consent forms) will be stored on University of Plymouth OneDrive as access requires a university username and password. Backups will be made on a University staff computer hard disk drive as these are located on University laptops that require a username a password. All data only be accessed by project staff, and will be anonymised and only be identified by a study ID number. Name and study ID numbers will be stored securely on a separate tracking sheet. All data will be archived for 10 years following study completion. On completion of the 10 year archive period, and following confirmation from the sponsor and CI, all digital data will be destroyed.

Patient and Public involvement

PwP and their care partners have been represented in the project group and have been since the project's inception. They have contributed to website and study design and will be providing feedback to finalise the design of the platform prior to beta-testing. They have been and will continue to be responsible for contributing to all patient and public facing materials relating to the project and the dissemination of its findings.

ETHICS AND DISSEMINATION

The University of Plymouth Faculty of Health Research Ethics and Integrity Committee (Ref. 3291) approved the use of interviews to capture usability feedback on 3rd May 2022. The university will also act as project sponsor.

Any protocol modifications will be reported on the website. The project team will prepare a plain English summary of the usability evaluation results which will also be displayed on the website and sent to the users who took part in beta-testing. The final results of the project will be disseminated via presentations at appropriate scientific meetings and conferences and publication in appropriate peer-reviewed journals, as well as dissemination within the Parkinson's patient community.

DISCUSSION

The aim of the Parkinson's research platform is to increase the communication and participation of PwP in clinical research and to reduce the administrative burden involved in enabling this participation. There is an urgent need to address the challenge of recruiting PwP to research studies. Web-based platforms can increase the efficiency of recruitment to PD studies, helping to ensure that recruitment targets are met within planned timeframes. This usability project undertakes robust usability evaluation of a new online research matching platform, something that has not previously been created specifically for PwP.

Inclusivity of participants is a particular issue in PD studies, particularly in terms of age, social deprivation, gender and ethnicity (20), which has a major effect on the generalisability of trial findings. The NIHR-INCLUDE project highlights the multidimensional and intersectional nature of the inclusion of under-served groups, and defines examples of potential barriers, such as a lack of

For peer review only - http://bmjopen.bmj.com/site/about/guidelines.xhtml

available trials and poor trial promotion (21). We expect our web-based tool to support inclusivity; we will therefore ensure that our evaluation covers the breadth of the workforce and potential participants with regard to geographical location, demographics, ethnicity, and socioeconomic factors. Digital literacy will also be captured as this been identified as a potential challenge in the use of digital technologies within healthcare (22). Furthermore, it is an important factor to capture in online usability assessments, particularly those involving older adults, as level of education or digital literacy are likely to influence how the user perceives the usability of the platform (23).

Developing digital solutions for older adults with Parkinson's presents specific challenges related to both age and disease, and so these need to be considered so that the platform matches the users' needs and characteristics. By using the MOLD-US framework to inform the interview topic guide it allows these challenges to be addressed. The framework has been previously used to assess usability barriers in older adults in the evaluation of health technologies in other disease areas and to allow for broader representation of the general ageing population (24).

The framework identifies four key categories of ageing barriers which influence the usability of health technologies, which are of particular relevance in PD:

1. Cognition - as one of the most common non-motor features of PD (25), cognitive impairment can influence memory, processing speed and attention (26).
2. Physical ability - PD is characterised by motor impairments such as bradykinesia, muscular rigidity and tremor (27). Slower movements and tremor may impact the speed of performance and increase error rate, leading to less subjective satisfaction (24).
3. Perception - visual impairments in PD include factors such as colour perception; the visual effectiveness of certain colours can therefore compromise usability performance (28).
4. Motivation - Up to 70% of PwP experience apathy (29), resulting in reduced interest and initiative, If the perceived value and ease of use of a technology interface is not immediate, then older adults are much less likely to use it in the future (30).

There have been misconceptions reported by research teams for other online platforms such as Join Dementia Research (JDR), including location of website users, a lack of awareness in contacting potential participants and the context of where the platform sits in the wider NHS recruitment landscape (31). Therefore, to ensure all users and stakeholders have full understanding of the platform's performance and functionality, as well as enabling platform optimisation as a clinical Parkinson's research trial recruitment tool in the future, the capture of Key Performance Indicators (KPI) such as national uptake, inclusivity and recruitment performance over time will be crucial.

This evaluation has important implications for the availability of research opportunities for PwP, by maximising the functionality and accessibility of an online platform that is tailored to the needs of both patients and study staff. It also has the potential to increase the efficiency of recruitment to PD studies, helping to ensure that recruitment targets are met for interventional trials within their planned timeframes. As a UK-wide platform, it will also support inclusivity in trial recruitment, thereby facilitating more representative and generalisable trial results.

ACKNOWLEDGEMENTS

We would like to thank the additional patient and care partner volunteers who gave input to the design of the usability methodology, and Dr Lexy Sorrell who assisted with sample size calculation.

For peer review only - http://bmjopen.bmj.com/site/about/guidelines.xhtml

COMPETING INTERESTS

None declared

FUNDING STATEMENT

This work was supported by unrestricted grants from Roche, Pfizer and Lundbeck. The funders did not contribute to the protocol in any way.

AUTHORS' CONTRIBUTIONS

CC, MLZ, EM, SM and RC conceived the usability project design, and SW, JW, KH and PH had further input into the design of the usability methodology. RC wrote all the drafts of the protocol with significant input from CC and MLZ. EM, SM, SW, JW, KH and PH reviewed and revised the manuscript. All authors approved the final manuscript as submitted.

REFERENCES

1. Vaswani PA, Tropea TF, Dahodwala N. Overcoming Barriers to Parkinson Disease Trial Participation: Increasing Diversity and Novel Designs for Recruitment and Retention. Neurotherapeutics. 2020;17(4):1724-35.
2. Podlewska AM, van Wamelen DJ. Parkinson's disease and Covid-19: The effect and use of telemedicine. Int Rev Neurobiol. 2022;165:263-81.
3. Picillo M, Kou N, Barone P, Fasano A. Recruitment strategies and patient selection in clinical trials for Parkinson's disease: Going viral and keeping science and ethics at the highest standards. Parkinsonism Relat Disord. 2015;21(9):1041-8.
4. Treweek S, Pitkethly M, Cook J, Fraser C, Mitchell E, Sullivan F, et al. Strategies to improve recruitment to randomised trials. Cochrane Database of Systematic Reviews. 2018;2018(2).
5. Carroll C, Palmer A, Cosby C, Zajicek J. Analysis and validation of a Parkinson's disease register as a recruitment tool for clinical studies. Clinical Medicine. 2012;12(3):210-5.
6. JDR. [Available from: https://www.joindementiaresearch.nihr.ac.uk/.
7. Kotting P, Smith A, O'Hare MB, Giebel C, Mendis L, Shaw C, et al. A national open-access research registry to improve recruitment to clinical studies. Alzheimers Dement (N Y). 2021;7(1):e12221.
8. Calculator I. [Available from: https://imd-by-postcode.opendatacommunities.org/imd/2019.
9. IMD W. [Available from: https://statswales.gov.wales/Catalogue/Community-Safety-and-Social-Inclusion/Welsh-Index-of-Multiple-Deprivation.
10. IMD S. [Available from: https://www.gov.scot/collections/scottish-index-of-multiple-deprivation-2020/.
11. IMD NI. [Available from: https://www.nisra.gov.uk/statistics/deprivation/northern-ireland-multiple-deprivation-measure-2017-nimdm2017.
12. Bank L. [Available from: https://www.lloydsbank.com/assets/media/pdfs/banking_with_us/whats-happening/LB-Consumer-Digital-Index-2018-Report.pdf.
13. Parmanto B, Lewis JAN, Graham KM, Bertolet MH. Development of the Telehealth Usability Questionnaire (TUQ). International Journal of Telerehabilitation. 2016;8(1):3-10.
14. Wildenbos GA, Peute L, Jaspers M. Aging barriers influencing mobile health usability for older adults: A literature based framework (MOLD-US). Int J Med Inform. 2018;114:66-75.
15. HotJar.
16. Daniel WW, Cross CL. Biostatistics: A Foundation for Analysis in the Health Sciences, 7th Edition. New York: John Wiley & Sons 2018.
17. Green J, Thorogood N. Qualitative Methods for Health Research: London: SAGE 2018.

For peer review only - http://bmjopen.bmj.com/site/about/guidelines.xhtml

18. Sauro J, Lewis JR. Quantifying the User Experience: Practical Statistics for User Research: Morgan Kaufmann; 2016 2016/7/12. 350 p.
19. Braun V, Clarke V. Using thematic analysis in psychology. Qual Res Psychol. 2006;3(2):77-101.
20. Dobkin RD, Amondikar N, Kopil C, Caspell-Garcia C, Brown E, Chahine LM, et al. Innovative Recruitment Strategies to Increase Diversity of Participation in Parkinson's Disease Research: The Fox Insight Cohort Experience. J Parkinsons Dis. 2020;10(2):665-75.
21. NIHR.
22. Topol E. The Topol review: preparing the healthcare workforce to deliver the digital future. Health Education England.
23. Silva AG, Caravau H, Martins A, Almeida AMP, Silva T, Ribeiro Ó, et al. Procedures of User-Centered Usability Assessment for Digital Solutions: Scoping Review of Reviews Reporting on Digital Solutions Relevant for Older Adults. JMIR Hum Factors. 2021;8(1):e22774-e.
24. Engelsma T, Jaspers MWM, Peute LW. Considerate mHealth design for older adults with Alzheimer's disease and related dementias (ADRD): A scoping review on usability barriers and design suggestions. Int J Med Inform. 2021;152:104494.
25. Maggio MG, Luca A, D'Agate C, Italia M, Calabrò RS, Nicoletti A. Feasibility and usability of a non-immersive virtual reality tele-cognitive app in cognitive rehabilitation of patients affected by Parkinson's disease. Psychogeriatrics. 2022;22(6):775-9.
26. Martins AI, Silva AG, Pais J, Cruz VT, Rocha NP. The impact of users' cognitive function on evaluator perceptions of usability. Sci Rep. 2022;12(1):13753.
27. Kalia LV, Lang AE. Parkinson's disease. Lancet. 2015;386(9996):896-912.
28. Harte R, Glynn L, Broderick B, Rodriguez-Molinero A, Baker P, McGuiness B, et al. Human Centred Design Considerations for Connected Health Devices for the Older Adult. Journal of Personalized Medicine. 2014;4(2):245-81.
29. Mele B, Van S, Holroyd-Leduc J, Ismail Z, Pringsheim T, Goodarzi Z. Diagnosis, treatment and management of apathy in Parkinson's disease: a scoping review. BMJ Open. 2020;10(9):e037632.
30. Matthew-Maich N, Harris L, Ploeg J, Markle-Reid M, Valaitis R, Ibrahim S, et al. Designing, Implementing, and Evaluating Mobile Health Technologies for Managing Chronic Conditions in Older Adults: A Scoping Review. JMIR mHealth and uHealth. 2016;4(2):e29.
31. Karagiannidou M, Stevens M, Knapp M, Cyhlarova E. Recruitment into dementia studies: Experiences of researchers using the Join Dementia Research register. Int J Geriatr Psychiatry. 2022;37(1):n/a.

Figure 1: Project Milestones

Figure 2: Website Functionality
[a]Tasks completed by the project Administrator on a separate account

[β]Study screening is performed externally to the website

[γ]Timepoint website user is invited to take part in an interview

For peer review only

For peer review only - http://bmjopen.bmj.com/site/about/guidelines.xhtml

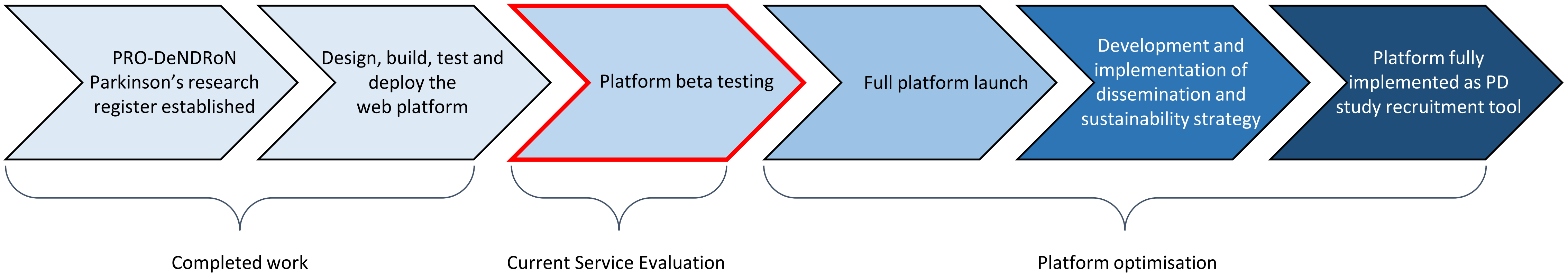
PRO-DeNDRoN Parkinson's research register established
Design, build, test and deploy the web platform
Platform beta testing
Full platform launch
Development and implementation of dissemination and sustainability strategy
Platform fully implemented as PD study recruitment tool
Completed work
Current Service Evaluation
Platform optimisation

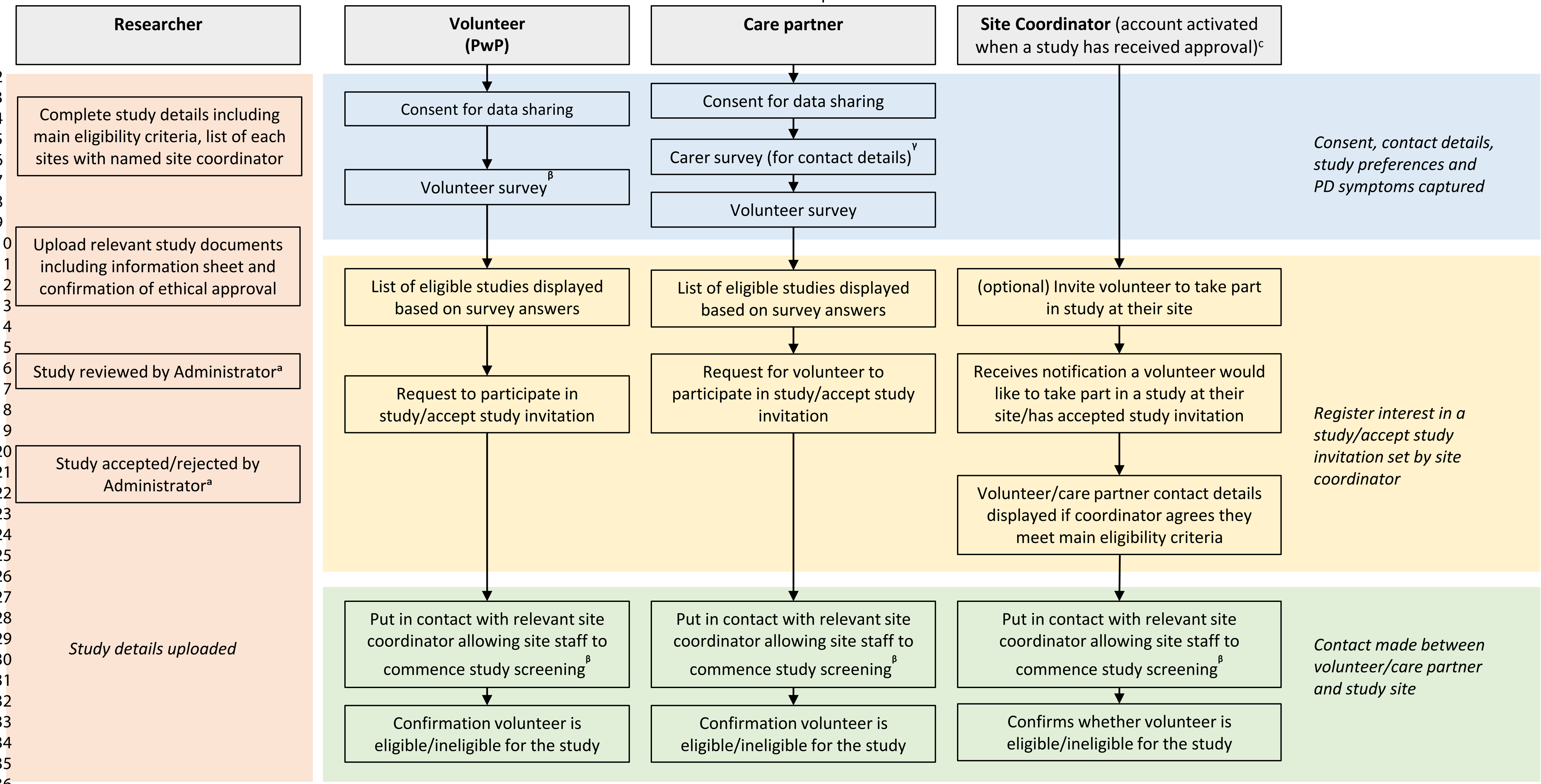
Researcher
Volunteer (PwP)
Care partner
Site Coordinator (account activated when a study has received approval)[c]
Complete study details including main eligibility criteria, list of each sites with named site coordinator
Upload relevant study documents including information sheet and confirmation of ethical approval
Study reviewed by Administrator[a]
Study accepted/rejected by Administrator[a]
Study details uploaded
Consent for data sharing
Volunteer survey[β]
Consent for data sharing
Carer survey (for contact details)[γ]
Volunteer survey
Consent, contact details, study preferences and PD symptoms captured
List of eligible studies displayed based on survey answers
Request to participate in study/accept study invitation
List of eligible studies displayed based on survey answers
Request for volunteer to participate in study/accept study invitation
(optional) Invite volunteer to take part in study at their site
Receives notification a volunteer would like to take part in a study at their site/has accepted study invitation
Volunteer/care partner contact details displayed if coordinator agrees they meet main eligibility criteria
Register interest in a study/accept study invitation set by site coordinator
Put in contact with relevant site coordinator allowing site staff to commence study screening[β]
Confirmation volunteer is eligible/ineligible for the study
Put in contact with relevant site coordinator allowing site staff to commence study screening[β]
Confirmation volunteer is eligible/ineligible for the study
Put in contact with relevant site coordinator allowing site staff to commence study screening[β]
Confirms whether volunteer is eligible/ineligible for the study
Contact made between volunteer/care partner and study site

Group 1 (PwP/Care partners) Interview Registration Form [to be inserted into JISC survey)

Section 1

The following data is collected so that we are able to contact you to arrange an interview:

First Name:

Surname:

Date of Birth:

Email Address:

Phone Number:

Preferred method of contact:

- Email
- Phone

Section 2

We would like to ensure that the JPR website is as user friendly as possible for everyone. By telling us a little bit more about yourself, you will help us achieve this goal.

Post Code:

Gender

Please select your gender

- Male
- Female
- Other (please specify)

Ethnicity

Please select your ethnicity:

- Asian/Asian British – Indian
- Asian/Asian British – Pakistani
- Asian/Asian British – Bangladeshi
- Asian/Asian British – Chinese
- Asian/Asian British – Any other Asian Background
- Black/African/Caribbean Background – African
- Black/African/Caribbean Background – Caribbean
- Black/African/Caribbean Background – Any other Black/African/Caribbean background
- Mixed Ethnic Group – White and Black Caribbean
- Mixed Ethnic Group – White and Black African
- Mixed Ethnic Group – White and Asian
- Mixed Ethnic Group – Any other mixed ethnic group
- Other Ethnic Group – Arab
- Other Ethnic Group – Berber Arab
- Other Ethnic Group – Ashkenazi Jewish
- White – English/Welsh/Scottish/Northern Irish/British
- White – Irish

For peer review only

- White – Gypsy or Irish Traveller
- White – Any other background

Parkinson's disease diagnosis (not applicable for Care Partners)

- When were you diagnosed with Parkinson's disease? (please give an approximate date if you are not sure) (dd/mm/yyyy)

Familiarity with digital technology

Please indicate if you are able to do any of the following tasks on a computer, phone or tablet

Managing information

- Use a search engine to look for information online
- Find a website I have visited before
- Download/save a photo I found online

Communicating

- Send a personal message via email or online messaging service
- Carefully make comments and share information online

Transacting

- Buy items or services from a website
- Buy and install apps on a device

Problem solving

- Verify sources of information I found online
- Solve a problem with a device/digital service using online help

Creating

- Complete online application forms which include personal details
- Create something new from existing online images, music or video

For peer review only

Group 2 (Site Coordinators) Interview Registration Form (to be inserted into JISC survey)

Section 1

The following data is collected so that we are able to contact you to arrange an interview:

First name:
Surname:
Date of birth:
Phone number:
Email address:
Site name:
Site Post Code:
Study name:
Preferred method of contact:

- Email
- Phone

Section 2

We would like to ensure that the JPR website is as user friendly as possible for everyone. By telling us a little bit more about yourself, you will help us achieve this goal.

Please tell us how you have been using JPR

- As a researcher
- As a site coordinator
- As both a researcher and site coordinator

Gender

Please select your gender

- Male
- Female
- Other (please specify)

Ethnicity

Please select your ethnicity:

- Asian/Asian British – Indian
- Asian/Asian British – Pakistani
- Asian/Asian British – Bangladeshi
- Asian/Asian British – Chinese
- Asian/Asian British – Any other Asian Background
- Black/African/Caribbean Background – African
- Black/African/Caribbean Background – Caribbean
- Black/African/Caribbean Background – Any other Black/African/Caribbean background

For peer review only

- Mixed Ethnic Group – White and Black Caribbean
- Mixed Ethnic Group – White and Black African
- Mixed Ethnic Group – White and Asian
- Mixed Ethnic Group – Any other mixed ethnic group
- Other Ethnic Group – Arab
- Other Ethnic Group – Berber Arab
- Other Ethnic Group – Ashkenazi Jewish
- White – English/Welsh/Scottish/Northern Irish/British
- White – Irish
- White – Gypsy or Irish Traveller
- White – Any other background

For peer review only

What type of Trust is your site a part of?
Please select the most relevant Trust type

- Large Acute
- Medium Acute
- Small Acute
- Mental Health
- Other (please specify)
- Not known

How many Parkinson's disease studies have you coordinated?

- Please specify the number of Parkinson's disease studies you have coordinated at your current site, and any previous sites you have worked at. If you are not sure, please give a rough estimate.

Familiarity with digital technology

Please indicate if you are able to do any of the following tasks on a computer, phone or tablet

Managing information

- Use a search engine to look for information online
- Find a website I have visited before
- Download/save a photo I found online

Communicating

- Send a personal message via email or online messaging service
- Carefully make comments and share information online

Transacting

- Buy items or services from a website
- Buy and install apps on a device

Problem solving

- Verify sources of information I found online
- Solve a problem with a device/digital service using online help

Creating

- Complete online application forms which include personal details
- Create something new from existing online images, music or video

For peer review only

Group 1 (volunteers/care partners) Prototype Topic Guide for Interviews

Interviewer introduces themselves

Before we start, is it okay with you if I audio-record this session?

- The reason we record is so we can go back and remember what was said and what wasn't said. Destroyed as soon as study is done, any publications don't use names
    - *Ask again for confirmation once recording is on*
    - If no: "That's fine, is it okay if I type notes as we talk and share those notes with you once we have finished the interview so you can make sure they are accurate?"

The purpose of this study is find out about your experiences of using the Join Parkinson's disease website, so we can find out what is working well or what needs to be changed or improved. Your feedback is very valuable for us to evaluate the website.

Today we're going to be going through some questions to discuss your experiences using the website, and then you will have the opportunity to provide feedback on anything else you want to talk about that you feel we haven't covered. If there is anything you don't understand during the interview, feel free to ask!

Before we get into those questions, I just wanted to remind you that all of your answers will be kept confidential and stored on a password-protected computer that only the research team can access. The audio recording that we're taking today will be deleted as soon as it has been transcribed, and any identifying information (like your name) will be removed from the audio recording before it is transcribed.

So before we get started, do you have any questions?

1. General usability

- How would you describe your experience of using the Join Parkinson's Research website?
    - *How much did you like or dislike using the website? (Why was this? What made you like/dislike that aspect?)*
    - *How easy or difficult did you find the website to use? (Why was this? What made that aspect easy or difficult to use?)*

2. Registration and survey completion

- How did you find the process of registering for the website?
    - Do you feel there is anything that could be improved with the registration process?
    - If so, what?
- [PwP only] How did you find the demographic survey?
- [Carer only] How did you find the volunteer survey completion?
    - [Both] Were there any questions that you found difficult?

For peer review only

3. Finding studies

- Did you find it easy to find studies that you might be eligible for?
    - If no, why not?
    - If yes, what was your experience of this?
- What did you think of the study information provided?
    - Is there any other information about a study that you would want to know?
- Did you feel there was anything that could be improved when searching for studies?
    - If so, what?

4. Study enrolment and researcher contact

- Have you enrolled on to any studies through JPR?
    - If yes, what did you find easy about the process?
    - What did you find tricky?
    - Could anything have been clearer?
    - If no, why not?

5. General feedback

- What would you suggest to make the website better?
    - *Can you tell me more about that, and how you think it would improve the website?*
    - *Are there any other suggestion that you think would make the website better?*
- Is there anything else you would like to mention?

For peer review only

Group 2 (site coordinators) Prototype Topic Guide for Interviews

Interviewer introduces themselves

Before we start, is it okay with you if I audio-record this session?

- The reason we record is so we can go back and remember what was said and what wasn't said. Destroyed as soon as study is done, any publications don't use names
  - *Ask again for confirmation once recording is on*
  - If no: "That's fine, is it okay if I type notes as we talk and share those notes with you once we have finished the interview so you can make sure they are accurate?"

The purpose of this study is find out about your experiences of using the Join Parkinson's disease website, so we can find out what is working well or what needs to be changed or improved. Your feedback is very valuable for us to evaluate the website.

Today we're going to be going through some questions to discuss your experiences using the website, and then you will have the opportunity to provide feedback on anything else you want to talk about that you feel we haven't covered. If there is anything you don't understand during the interview, feel free to ask!

Before we get into those questions, I just wanted to remind you that all of your answers will be kept confidential and stored on a password-protected computer that only the research team can access. The audio recording that we're taking today will be deleted as soon as it has been transcribed, and any identifying information (like your name) will be removed from the audio recording before it is sent to the transcription service.

So before we get started, do you have any questions?

1. **General usability**

- How would you describe your experience of using the Join Parkinson's Research website?
  - *How much did you like or dislike using the website?(Why was this? What made you like/dislike that aspect?)*
  - *How easy or difficult did you find the website to use? (Why was this? What made that aspect easy or difficult to use?)*

2. **Registration**

- How did you find the process of registering for the website?
  - Do you feel there is anything that could be improved in terms of being registered as a site study coordinator for your study? If so, what?

3. **Inviting and enrolling participants**

- Did any volunteers send a request to participate in your study?
  - Have they been enrolled onto the study?
  - If no, why not?
- Did you invite any volunteers to enrol on your study?

For peer review only

- If no, why not?

- Once a volunteer had requested participation, or accepted the study invite, what did you find easy about the enrolment process?
  - What made it tricky?
  - Could anything have been clearer?
  - Did you feel there was anything else that could be improved? If so, what?

4. General feedback

- What would you suggest to make the website better/easier to use/easier to navigate/functions that you would like to see?
  - *Can you tell me more about that, and how you think it would improve the website?*
  - *Are there any other suggestion that you think would make the website better?*
- Is there anything else you would like to mention?

For peer review only

STANDARD PROTOCOL ITEMS: RECOMMENDATIONS FOR INTERVENTIONAL TRIALS

SPIRIT 2013 Checklist: Recommended items to address in a clinical trial protocol and related documents*

Section/item	Item No	Description	Addressed on page number
**Administrative information**			
Title	1	Descriptive title identifying the study design, population, interventions, and, if applicable, trial acronym	_____1_______
Trial registration	2a	Trial identifier and registry name. If not yet registered, name of intended registry	n/a. This project is an evaluation and will not be registered
	2b	All items from the World Health Organization Trial Registration Data Set	_____n/a______
Protocol version	3	Date and version identifier	n/a. This is a project methods manuscript
Funding	4	Sources and types of financial, material, and other support	______9______
Roles and responsibilities	5a	Names, affiliations, and roles of protocol contributors	_____1,9______
	5b	Name and contact information for the trial sponsor	_____7_______
	5c	Role of study sponsor and funders, if any, in study design; collection, management, analysis, and interpretation of data; writing of the report; and the decision to submit the report for publication, including whether they will have ultimate authority over any of these activities	_____7,9______

For peer review only - http://bmjopen.bmj.com/site/about/guidelines.xhtml

	5d	Composition, roles, and responsibilities of the coordinating centre, steering committee, endpoint adjudication committee, data management team, and other individuals or groups overseeing the trial, if applicable (see Item 21a for data monitoring committee)	n/a. This is an evaluation project
**Introduction**			
Background and rationale	6a	Description of research question and justification for undertaking the trial, including summary of relevant studies (published and unpublished) examining benefits and harms for each intervention	____2,3_______
	6b	Explanation for choice of comparators	n/a. This usability evaluation will not compare different groups
Objectives	7	Specific objectives or hypotheses	______3______
Trial design	8	Description of trial design including type of trial (eg, parallel group, crossover, factorial, single group), allocation ratio, and framework (eg, superiority, equivalence, noninferiority, exploratory)	______3______
**Methods: Participants, interventions, and outcomes**			
Study setting	9	Description of study settings (eg, community clinic, academic hospital) and list of countries where data will be collected. Reference to where list of study sites can be obtained	_____3______
Eligibility criteria	10	Inclusion and exclusion criteria for participants. If applicable, eligibility criteria for study centres and individuals who will perform the interventions (eg, surgeons, psychotherapists)	_____4,5______
Interventions	11a	Interventions for each group with sufficient detail to allow replication, including how and when they will be administered	_____4,5,6____
	11b	Criteria for discontinuing or modifying allocated interventions for a given trial participant (eg, drug dose change in response to harms, participant request, or improving/worsening disease)	n/a. This project does not test an intervention

For peer review only

	11c	Strategies to improve adherence to intervention protocols, and any procedures for monitoring adherence (eg, drug tablet return, laboratory tests)	n/a. This project does not test an intervention
	11d	Relevant concomitant care and interventions that are permitted or prohibited during the trial	n/a. This project does not test an intervention
Outcomes	12	Primary, secondary, and other outcomes, including the specific measurement variable (eg, systolic blood pressure), analysis metric (eg, change from baseline, final value, time to event), method of aggregation (eg, median, proportion), and time point for each outcome. Explanation of the clinical relevance of chosen efficacy and harm outcomes is strongly recommended	_____5,6______
Participant timeline	13	Time schedule of enrolment, interventions (including any run-ins and washouts), assessments, and visits for participants. A schematic diagram is highly recommended (see Figure)	Figure 2
Sample size	14	Estimated number of participants needed to achieve study objectives and how it was determined, including clinical and statistical assumptions supporting any sample size calculations	______6______
Recruitment	15	Strategies for achieving adequate participant enrolment to reach target sample size	_____3,4______

Methods: Assignment of interventions (for controlled trials)

Allocation:

Sequence generation	16a	Method of generating the allocation sequence (eg, computer-generated random numbers), and list of any factors for stratification. To reduce predictability of a random sequence, details of any planned restriction (eg, blocking) should be provided in a separate document that is unavailable to those who enrol participants or assign interventions	4 (explains purposive sampling for interviews
Allocation concealment mechanism	16b	Mechanism of implementing the allocation sequence (eg, central telephone; sequentially numbered, opaque, sealed envelopes), describing any steps to conceal the sequence until interventions are assigned	n/a

For peer review only - http://bmjopen.bmj.com/site/about/guidelines.xhtml

Implementation	16c	Who will generate the allocation sequence, who will enrol participants, and who will assign participants to interventions	4 (explains purposive sampling for interviews
Blinding (masking)	17a	Who will be blinded after assignment to interventions (eg, trial participants, care providers, outcome assessors, data analysts), and how	n/a
	17b	If blinded, circumstances under which unblinding is permissible, and procedure for revealing a participant's allocated intervention during the trial	n/a
**Methods: Data collection, management, and analysis**			
Data collection methods	18a	Plans for assessment and collection of outcome, baseline, and other trial data, including any related processes to promote data quality (eg, duplicate measurements, training of assessors) and a description of study instruments (eg, questionnaires, laboratory tests) along with their reliability and validity, if known. Reference to where data collection forms can be found, if not in the protocol	______5,6_____
	18b	Plans to promote participant retention and complete follow-up, including list of any outcome data to be collected for participants who discontinue or deviate from intervention protocols	n/a. There will only be one assessment timepoint for each participant
Data management	19	Plans for data entry, coding, security, and storage, including any related processes to promote data quality (eg, double data entry; range checks for data values). Reference to where details of data management procedures can be found, if not in the protocol	_____5,6,7_____
Statistical methods	20a	Statistical methods for analysing primary and secondary outcomes. Reference to where other details of the statistical analysis plan can be found, if not in the protocol	_____6_______
	20b	Methods for any additional analyses (eg, subgroup and adjusted analyses)	n/a. No additional analysis will be undertaken

	20c	Definition of analysis population relating to protocol non-adherence (eg, as randomised analysis), and any statistical methods to handle missing data (eg, multiple imputation)	n/a. All survey questions will be mandatory
**Methods: Monitoring**			
Data monitoring	21a	Composition of data monitoring committee (DMC); summary of its role and reporting structure; statement of whether it is independent from the sponsor and competing interests; and reference to where further details about its charter can be found, if not in the protocol. Alternatively, an explanation of why a DMC is not needed	_____7_______
	21b	Description of any interim analyses and stopping guidelines, including who will have access to these interim results and make the final decision to terminate the trial	n/a. This is an evaluation project
Harms	22	Plans for collecting, assessing, reporting, and managing solicited and spontaneously reported adverse events and other unintended effects of trial interventions or trial conduct	n/a. This is an evaluation project
Auditing	23	Frequency and procedures for auditing trial conduct, if any, and whether the process will be independent from investigators and the sponsor	n/a. This is an evaluation project
**Ethics and dissemination**			
Research ethics approval	24	Plans for seeking research ethics committee/institutional review board (REC/IRB) approval	______7______
Protocol amendments	25	Plans for communicating important protocol modifications (eg, changes to eligibility criteria, outcomes, analyses) to relevant parties (eg, investigators, REC/IRBs, trial participants, trial registries, journals, regulators)	______7______
Consent or assent	26a	Who will obtain informed consent or assent from potential trial participants or authorised surrogates, and how (see Item 32)	______4______
	26b	Additional consent provisions for collection and use of participant data and biological specimens in ancillary studies, if applicable	n/a

For peer review only - http://bmjopen.bmj.com/site/about/guidelines.xhtml

Confidentiality	27	How personal information about potential and enrolled participants will be collected, shared, and maintained in order to protect confidentiality before, during, and after the trial	_____5,6,7_____
Declaration of interests	28	Financial and other competing interests for principal investigators for the overall trial and each study site	______9_______
Access to data	29	Statement of who will have access to the final trial dataset, and disclosure of contractual agreements that limit such access for investigators	______7_______
Ancillary and post-trial care	30	Provisions, if any, for ancillary and post-trial care, and for compensation to those who suffer harm from trial participation	n/a. This project does not test an intervention
Dissemination policy	31a	Plans for investigators and sponsor to communicate trial results to participants, healthcare professionals, the public, and other relevant groups (eg, via publication, reporting in results databases, or other data sharing arrangements), including any publication restrictions	______7______
	31b	Authorship eligibility guidelines and any intended use of professional writers	not provided
	31c	Plans, if any, for granting public access to the full protocol, participant-level dataset, and statistical code	_____n/a______
**Appendices**			
Informed consent materials	32	Model consent form and other related documentation given to participants and authorised surrogates	not provided
Biological specimens	33	Plans for collection, laboratory evaluation, and storage of biological specimens for genetic or molecular analysis in the current trial and for future use in ancillary studies, if applicable	_____n/a______

*It is strongly recommended that this checklist be read in conjunction with the SPIRIT 2013 Explanation & Elaboration for important clarification on the items. Amendments to the protocol should be tracked and dated. The SPIRIT checklist is copyrighted by the SPIRIT Group under the Creative Commons "Attribution-NonCommercial-NoDerivs 3.0 Unported" license.

Incorporating usability evaluation into iterative development of an online platform to support research participation in Parkinson’s disease: A mixed methods protocol

Journal:	*BMJ Open*
Manuscript ID	bmjopen-2023-078638.R1
Article Type:	Protocol
Date Submitted by the Author:	29-Sep-2023
Complete List of Authors:	Chapman, Rebecca; University of Plymouth, Applied Parkinson's Research Group, Faculty of Health<br>Zeissler, Marie-Louise; University of Plymouth, Applied Parkinson's Research Group, Faculty of Health<br>Meinert, Edward; University of Plymouth, Centre for Health Technology, Faculty of Health ; Newcastle University, Translational and Clinical Research Institute<br>Mullin, Stephen; University of Plymouth, Applied Parkinson's Research Group, Faculty of Health<br>Whipps, Sue; University of Plymouth, Patient and Public Involvement (PPI) representative<br>Whipps, John; University of Plymouth, Patient and Public Involvement (PPI) representative<br>Hockey, Kate; University of Plymouth, Patient and Public Involvement (PPI) representative<br>Hockey, Philip; University of Plymouth, Patient and Public Involvement (PPI) representative<br>Carroll, Camille; University of Plymouth, Applied Parkinson's Research Group, Faculty of Medicine and Dentistry; Newcastle University, Translational and Clinical Research Institute
**Primary Subject Heading**:	Research methods
Secondary Subject Heading:	Neurology, Qualitative research
Keywords:	Parkinson-s disease < NEUROLOGY, QUALITATIVE RESEARCH, REGISTRIES, Surveys and Questionnaires

SCHOLARONE™ Manuscripts

Incorporating usability evaluation into iterative development of an online platform to support research participation in Parkinson's disease: A mixed methods protocol

Authors: Rebecca Chapman[1], Marie-Louise Zeissler[1], Edward Meinert[2, 3, 4], Stephen Mullin[1], Sue Whipps[5], John Whipps[5], Kate Hockey[5], Phil Hockey[5], Camille Carroll[1, 4]

Affiliations:

[1] Applied Parkinson's Research Group, University of Plymouth, Faculty of Health, Plymouth, United Kingdom; [2]Centre for Health Technology, University of Plymouth, Faculty of Health, Plymouth, United Kingdom; [3]Primary Care and Public Health, Imperial College London, London, United Kingdom; [4]Translational and Clinical Research Institute, Newcastle University, Newcastle, UK;

[5]Patient and Public Involvement Representative

Corresponding Author: Professor Camille Carroll, Translational and Clinical Research Institute, Newcastle University, NE1 7RU, United Kingdom

Email: camille.carroll@newcastle.ac.uk

For peer review only

ABSTRACT

Introduction

Many people with Parkinson's (PwP) are not given the opportunity or do not have adequate access to participate in clinical research. To address this, we have co-developed with users an online platform that connects PwP to clinical studies in their local area. It enables site staff to communicate with potential participants and aims to increase the participation of the Parkinson's community in research. This protocol outlines the mixed methods study protocol for the usability testing of the platform.

Methods and Analysis

We will seek user input to finalise the platform's design, which will then be deployed in a limited launch for beta testing. The beta version will be used as a recruitment tool for up to 3 studies with multiple UK sites. Usability data will be collected from the three intended user groups: PwP, care partners acting on their behalf and site study coordinators. Usability questionnaires and website analytics will be used to capture user experience quantitatively, and a purposive sample of users will be invited to provide further feedback via semi-structured interviews. Quantitative data will be analysed using descriptive statistics, and a thematic analysis undertaken for interview data. Data from this study will inform future platform iterations.

Ethics and Dissemination

Ethical approval was obtained from the University of Plymouth (3291; 3rd May 2022). We will share our findings via a 'Latest News' section within the platform, presentations, conference meetings and national PwP networks.

Strengths and Limitations of this Project

- A mixed methods approach will utilise both qualitative and quantitative methods and enhance our understanding of any usability issues identified in the development of the platform
- We will seek feedback on usability from recruiting staff at study sites as well as patients
- Purposive sampling for semi-structured interviews will ensure inclusivity in terms of demographics, geographical location, and digital literacy, to ensure issues are identified from a broad range of users
- Platform users may not have utilised the whole website prior to interview, and so their answers may not capture the entirety of the platform

INTRODUCTION

Delays in reaching recruitment targets represent a major challenge for clinical trials (1). A reduction in in-person clinic attendance with the introduction of new, remote care delivery models following

For peer review only - http://bmjopen.bmj.com/site/about/guidelines.xhtml

the COVID 19 pandemic has further exacerbated this problem (2). Parkinson's disease (PD) trials generally do not recruit representative populations and therefore their results are not generalisable, which risks perpetuating healthcare inequalities (3). Strategies to improve recruitment to trials have been evaluated, but other than telephone reminders and opt-out strategies, very few have been found to be effective (4). The creation of an online recruitment tool to facilitate communication with and recruitment of research-interested PD patients has the potential to increase the efficiency of recruitment to PD studies.

In 2007 we developed a paper-based register of research-interested PD patients within the South West of England. The Parkinson's Register of the Dementias and Neurodegenerative Diseases Research Network (PRO-DeNDRoN) has been previously evaluated, and recognised as a successful and resource efficient recruitment tool, with 85% of registered PRO-DeNDRoN recruiters reporting that the register was a useful means of facilitating research and providing data for planning of service provision (5). However, this required manual data entry resulting in administrative burden, and highlighted the need for a more efficient, online recruitment platform that is easily accessible to people with Parkinson's and time efficient for trial delivery staff.

Online platforms have been used in other disease areas as a successful means of trial recruitment. For example, 'Join Dementia Research' (JDR) is a website that connects people with dementia to research projects across the UK (6). The website has been established in 2015 and in December 2021 JDR reported to have a population of nearly 50,000 research-interested people registered, 12% of whom had a self-declared diagnosis of dementia, with 51% of these being women (7). This highlights the potential of online registers as a useful tool for disease-specific recruitment.

Originating from the PRO-DeNDRoN register, we have developed an online platform, conceptualised and designed with PwP and care partner input (see Figure 1 for project milestone overview). Our platform aims to connect people with Parkinson's (PwP) with research projects in their preferred geographical locations. The platform matches participants' eligibility and preferences to open studies and also enables site coordinators to communicate directly with potential participants. The platform has been designed with multi-account permissions in place: central study coordinators (known as 'researchers') who can request to upload a study, administrative staff who review the study documentation, PwP wishing to engage with research and care partners, who are able set up an account in order to register PwP for studies on their behalf, if the PwP does not wish to, or is not able to, use the platform themselves; care partners are not able to register for studies themselves. Care partners in this context are unpaid and are defined as "the primary person who feels responsible for, and supports, the PwP". There is also an account for study coordinators at each individual study site (created automatically once a study has been approved), who can engage with interested or eligible participants (see Figure 2 for an overview of website functionality).

This project aims to evaluate platform usability and accessibility in the three main user groups (PwP, care partners and study site coordinators), to understand barriers and facilitators to engagement and use.

METHODS

Project design

We will invite usability feedback from PwP, care partner and site coordinator representatives to identify any major bugs or functionality issues in the platform prior to beta testing. The platform will

For peer review only - http://bmjopen.bmj.com/site/about/guidelines.xhtml

then be deployed in a limited launch to enable mixed-methods evaluation via validated usability questionnaire, website analytics and semi-structured interviews.

Dissemination of platform beta-version

Prior to deployment, we will select up to 3 multi-centre studies in Parkinson's disease actively recruiting within the UK, which have good geographical spread of sites. We will invite research investigator teams to input their study details onto the platform. The beta-version of the platform will then be disseminated to the Parkinson's community via national patient and carer networks, as well as charity stakeholders, such as Parkinson's UK and Cure Parkinson's, which will include Steering Group meetings and advertisements through research information emails.

Study recruitment and participant selection

Usability questionnaires

All platform users will be invited to complete an online questionnaire which will be available to complete at any time. This will be visible as a tab on the user account menu (for the PwP and the care partner accounts) or an automated email following account activation (for site coordinators) where users can sign up separately for the questionnaire and semi-structured interviews. A link will divert users to Joint Information Systems Committee (JISC) online surveys, displaying an information sheet and e-consent form. A survey will then capture user type, ethnicity, age and socioeconomic status, followed by the usability questionnaire.

Website Analytics

Upon first visiting the platform, a pop-up will be displayed to ask if the user consents to the use of analytical website cookies. If the user consents, measurement of key performance indicators (KPIs) will be captured.

Semi-structured interviews

All users will be invited to register interest in participating in remotely conducted interviews to gain an understanding of how they experienced aspects of their user journey, as well as whether and how user experience could be improved. A separate question will be displayed on the same tab as for the usability questionnaires or automated email. For each group, the question will ask if they are interested in providing feedback via an interview and contain two links: one to the information sheet and one to the e-consent form. The information sheet will detail the purpose and nature of the interviews, information on what they involve, details regarding free choice and right to withdraw and will describe the retention of data provisions. The information sheet will be accessed via separate link to the consent form to allow users adequate time to consider the nature of the study and ask any questions they have. Once e-consent has been obtained, a JISC demographic survey will be displayed to facilitate purposive sampling (please see Supplementary Files 1 and 2 for a list of demographic questions).

Participants will be interviewed individually and purposively selected from the three intended platform user groups: PwP,care partners and researchers/site study coordinators. PwP and care partners will be selected based on ethnicity, socioeconomic status, disease duration (if applicable) and digital literacy. Researchers and site coordinators will be selected based on ethnicity, site type, PD research experience and digital literacy. If selected for interview, the participant will be contacted by a member of the project team via their preferred method indicated on the registration

For peer review only - http://bmjopen.bmj.com/site/about/guidelines.xhtml

survey and arrange a suitable time to conduct the interview. If not selected, the user will receive an email explaining this and thanking them for their time.

Inclusion criteria for all data collection methods

PwP/care partners

- Age ≥18 years
- Diagnosis of Parkinson's disease/ care partner of someone with PD (care partners in this context are unpaid and defined as "the primary person who feels responsible for, and supports, the PwP")
- Prior experience of using a computing device (including but not limited to PC, Mac & Tablet)
- Has access to a desktop or laptop computer with internet connection
- Willing and able to give informed consent for participation in the project
- Willing and able to comply with project requirements

Researchers/Site Study Coordinators

- Age ≥18 years
- Registered as a named central study coordinator and/or site study coordinator for a study registered on the platform
- Prior computer experience
- Willing and able to give informed consent for participation in the project
- Willing and able to comply with project requirements

The only specified exclusion criteria for all website users are being unable or unwilling to provide informed consent or comply with project requirements.

Data Collection

Demographic Information

Demographic data will be collected for all users who agree to complete the usability questionnaire and/or semi-structured interviews. The measurement tools selected will allow for purposive sampling and aim to maximise inclusivity of interview participants. Postcode will be collected to allow for capture of both rural and urban participants, as well as calculation of socioeconomic status via the Index of Multiple Deprivation (IMD) calculator (8). This tool provides the official measures of relative deprivation for small areas in England, and the equivalent tools will be used for the devolved nations (9-11). Digital literacy will be captured by reproducing the Lloyds Bank Basic Digital Skills Measure (2018) (12). This is a list of 11 digital tasks split over five skills categories. Respondents are classified as having full basic digital literacy skills if they can complete at least one task in each category.

Usability Questionnaires

Questionnaire feedback will be captured using the Telehealth Usability Questionnaire (TUQ) (13), which is suitable for the collection of opinions from both platform user groups (patients and clinical study site staff), and has previously been used in PD patients (14). It evaluates the usability of telehealth services and is based on six criteria including usefulness, ease of use and learnability, interface quality, interaction quality, reliability and satisfaction and future use. All questions are optional which allow the measure to the tailored to meet the requirements of specific digital services.

Semi-structured interviews

For peer review only - http://bmjopen.bmj.com/site/about/guidelines.xhtml

Semi-structured qualitative interviews will be conducted by a University of Plymouth researcher and take place either over the phone or via teleconferencing software and will last no longer than one hour. The participant will be facilitated through the interview process, and prompts will be used to enable guided conversations using an interview guide (see Supplementary Files 3 and 4 for prototype interview questions). This guide has been informed by the MOLD-US framework (15), which identifies four key categories of ageing barriers which influence the usability of health technologies, and that are of particular relevance in PD: cognition, physical ability, perception and motivation. This allows for results to be classified and interpreted based on impediments that are intrinsic to usability issues experienced by older adults. The topic guide will also be informed by the initial usability feedback prior to live deployment.

The guide contains questions that seek feedback on each aspect of website functionality, from user registration to confirmation of study eligibility (if applicable) and participants will be given the opportunity to identify what went well or what could be improved. All interview sessions will be recorded via video and/or audio capture and will be fully transcribed by a University of Plymouth researcher.

Website analytics

Key performance indicators (KPIs) of user behaviour will be measured using HotJar analytical software (16), where users consent for this to be captured through the acceptance of analytical website cookies. General user behaviour will be captured through the use of heatmaps, and individual user journeys will be mapped and navigation timings recorded.

Further performance indicators will include the number of PwP registered to the platform, the percentage requesting and/or invited to take part in a study, and the percentage of PwP accepted for study screening and enrolment.

Sample Size

TUQ

We determined a 95% confidence interval, an accuracy of +/-5 percentage units and a satisfaction with the website random estimation of 50%. Taking into account a target population limit of 500 participants that can be supported within the test-server the sample size needed is 218 participants (17).

Web analytics

We will gather data from all users who consent to the use of website analytics, but we will aim for 10 per user group, per data capture method.

Semi-structured interviews

A purposive sample of 20 users will be selected for semi-structured interviews (10 per participant group) will be selected. While still allowing for a richly textured understanding of the usability issues (18), it is maintained that little new information is generated after completing 20 qualitative interviews.

Data analysis

Quantitative data

For peer review only - http://bmjopen.bmj.com/site/about/guidelines.xhtml

Median (range) data will be collated on all usability questionnaire items as an indicator of usability levels for both user groups. For website analytics, average timings to complete user journey subtasks and individual recorded user journeys of “slow” and “fast” completers will be descriptively analysed to gain insights into processes that may cause difficulties. Feedback specific analysis will also be undertaken and depending on interview feedback, particular parts of website user journeys may be descriptively analysed further to gain insights into how optimal improvements can be made.

Qualitative data

For initial feedback prior to beta-testing, we will evaluate the risk of harm according to standard processes (19) which comprise an evaluation of task criticality, frequency and impact. Ratings with high severity of harm ratings will be prioritised for amendment over issues with less high ratings.

Interview data will be stored, managed and analysed with NVivo. Thematic analysis will be undertaken using the six-step approach of Braun and Clarke: 1) become familiar with data, 2) generate initial codes, 3) search for themes, 4) review themes, 5) define themes, 6) write-up (20).

Project Management

A project management team consisting of researchers, clinicians and PPI representatives will meet monthly and take on the role of data monitoring and project conduct.

All project data will be managed in line with local and national GDPR requirements. All digital data (including digital consent forms) will be stored on University of Plymouth OneDrive as access requires a university username and password. Backups will be made on a University staff computer hard disk drive as these are located on University laptops that require a username a password. All data only be accessed by project staff, and will be anonymised and only be identified by a study ID number. Name and study ID numbers will be stored securely on a separate tracking sheet. All data will be archived for 10 years following study completion. On completion of the 10 year archive period, and following confirmation from the sponsor and CI, all digital data will be destroyed.

Patient and Public involvement

PwP and their care partners have been represented in the project group and have been since the project’s inception. They have contributed to website and study design and will be providing feedback to finalise the design of the platform prior to beta-testing. They have been and will continue to be responsible for contributing to all patient and public facing materials relating to the project and the dissemination of its findings.

ETHICS AND DISSEMINATION

The University of Plymouth Faculty of Health Research Ethics and Integrity Committee (Ref. 3291) approved the use of interviews to capture usability feedback on 3rd May 2022. The university will also act as project sponsor.

Any protocol modifications will be reported on the website. The project team will prepare a plain English summary of the usability evaluation results which will also be displayed on the website and sent to the users who took part in beta-testing. The final results of the project will be disseminated via presentations at appropriate scientific meetings and conferences and publication in appropriate peer-reviewed journals, as well as dissemination within the Parkinson’s patient community.

DISCUSSION

For peer review only - http://bmjopen.bmj.com/site/about/guidelines.xhtml

The aim of the Parkinson's research platform is to increase the communication and participation of PwP in health and care research and to reduce the administrative burden involved in enabling this participation. There is an urgent need to address the challenge of recruiting PwP to research studies. Web-based platforms can increase the efficiency of recruitment to PD studies, helping to ensure that recruitment targets are met within planned timeframes. This usability project undertakes robust usability evaluation of a new online research matching platform, something that has not previously been created specifically for PwP.

Inclusivity of participants is a particular issue in PD studies, particularly in terms of age, social deprivation, gender and ethnicity (21), which has a major effect on the generalisability of trial findings. The NIHR-INCLUDE project highlights the multidimensional and intersectional nature of the inclusion of under-served groups, and defines examples of potential barriers, such as a lack of available trials and poor trial promotion (22). We expect our web-based tool to support inclusivity; we will therefore ensure that our evaluation covers the breadth of the workforce and potential participants with regard to geographical location, demographics, ethnicity, and socioeconomic factors. Digital literacy will also be captured as this been identified as a potential challenge in the use of digital technologies within healthcare (23). Furthermore, it is an important factor to capture in online usability assessments, particularly those involving older adults, as level of education or digital literacy are likely to influence how the user perceives the usability of the platform (24).

Although digital healthcare tools have been developed in conjunction with PwP (25), as well as those with other neurodegenerative diseases such as Alzheimer's (26), developing digital solutions for PwP, and in particular those that are older, still presents specific challenges related to both age and disease, and so these need to be considered so that the platform matches the users' needs and characteristics. By using the MOLD-US framework to inform the interview topic guide it allows these challenges to be addressed. The framework has been previously used to assess usability barriers in older adults in the evaluation of health technologies in other disease areas and to allow for broader representation of the general ageing population (26), and identifies the following key categories of ageing barriers which influence the usability of health technologies.

1. Cognition - as one of the most common non-motor features of PD (25), cognitive impairment can influence memory, processing speed and attention (27).
2. Physical ability - PD is characterised by motor impairments such as bradykinesia, muscular rigidity and tremor (28). Slower movements and tremor may impact the speed of performance and increase error rate, leading to less subjective satisfaction (26).
3. Perception - visual impairments in PD include factors such as colour perception; the visual effectiveness of certain colours can therefore compromise usability performance (29).
4. Motivation - Up to 70% of PwP experience apathy (30), resulting in reduced interest and initiative, If the perceived value and ease of use of a technology interface is not immediate, then older adults are much less likely to use it in the future (31).

There have been misconceptions reported by research teams for other online platforms such as Join Dementia Research (JDR), including location of website users, a lack of awareness in contacting potential participants and the context of where the platform sits in the wider NHS recruitment landscape (32). Therefore, to ensure all users and stakeholders have full understanding of the platform's performance and functionality, as well as enabling platform optimisation as a clinical Parkinson's research trial recruitment tool in the future, the capture of Key Performance Indicators

For peer review only - http://bmjopen.bmj.com/site/about/guidelines.xhtml

(KPI) such as national uptake, inclusivity and recruitment performance over time will be crucial.This evaluation has important implications for the availability of research opportunities for PwP, and our mixed methods approach will help to enhance the understanding of any usability issues identified in the development of the platform. Seeking feedback from recruiting staff at study sites as well as PwP and care partners will help maximise the functionality and accessibility of an online platform that is tailored to the needs of both patients and study staff. Purposive sampling for semi-structured interviews will ensure inclusivity in terms of demographics, geographical location, and digital literacy. However, by using a remote asynchronous method for evaluating usability we are not able to confirm whether each platform account is being used by a singular user and, in turn, their TUQ responses. Furthermore, platform users may not have utilised the whole website prior to interview, and so their answers may not capture entire platform functionality.

Our platform has the potential to increase the efficiency of recruitment to PD studies, helping to ensure that recruitment targets are met for interventional trials within their planned timeframes. As a UK-wide platform, it will also support inclusivity in trial recruitment, thereby facilitating more representative and generalisable trial results.

ACKNOWLEDGEMENTS

We would like to thank the additional patient and care partner volunteers who gave input to the design of the usability methodology, and Dr Lexy Sorrell who assisted with sample size calculation.

COMPETING INTERESTS

None declared

FUNDING STATEMENT

This work was supported by unrestricted grants from Roche, Pfizer and Lundbeck (award grant numbers n/a). The funders did not contribute to the protocol in any way.

AUTHORS' CONTRIBUTIONS

CC, MLZ, EM, SM and RC conceived the usability project design, and SW, JW, KH and PH had further input into the design of the usability methodology. RC wrote all the drafts of the protocol with significant input from CC and MLZ. EM, SM, SW, JW, KH and PH reviewed and revised the manuscript. All authors approved the final manuscript as submitted.

REFERENCES

1. Vaswani PA, Tropea TF, Dahodwala N. Overcoming Barriers to Parkinson Disease Trial Participation: Increasing Diversity and Novel Designs for Recruitment and Retention. Neurotherapeutics. 2020;17(4):1724-35.
2. Podlewska AM, van Wamelen DJ. Parkinson's disease and Covid-19: The effect and use of telemedicine. Int Rev Neurobiol. 2022;165:263-81.
3. Picillo M, Kou N, Barone P, Fasano A. Recruitment strategies and patient selection in clinical trials for Parkinson's disease: Going viral and keeping science and ethics at the highest standards. Parkinsonism Relat Disord. 2015;21(9):1041-8.
4. Treweek S, Pitkethly M, Cook J, Fraser C, Mitchell E, Sullivan F, et al. Strategies to improve recruitment to randomised trials. Cochrane Database of Systematic Reviews. 2018;2018(2).
5. Carroll C, Palmer A, Cosby C, Zajicek J. Analysis and validation of a Parkinson's disease register as a recruitment tool for clinical studies. Clinical Medicine. 2012;12(3):210-5.
6. JDR. [Available from: https://www.joindementiaresearch.nihr.ac.uk/.

For peer review only - http://bmjopen.bmj.com/site/about/guidelines.xhtml

7. Kotting P, Smith A, O'Hare MB, Giebel C, Mendis L, Shaw C, et al. A national open-access research registry to improve recruitment to clinical studies. Alzheimers Dement (N Y). 2021;7(1):e12221.
8. Calculator I. [Available from: https://imd-by-postcode.opendatacommunities.org/imd/2019.
9. IMD W. [Available from: https://statswales.gov.wales/Catalogue/Community-Safety-and-Social-Inclusion/Welsh-Index-of-Multiple-Deprivation.
10. IMD S. [Available from: https://www.gov.scot/collections/scottish-index-of-multiple-deprivation-2020/.
11. IMD NI. [Available from: https://www.nisra.gov.uk/statistics/deprivation/northern-ireland-multiple-deprivation-measure-2017-nimdm2017.
12. Bank L. [Available from: https://www.lloydsbank.com/assets/media/pdfs/banking_with_us/whats-happening/LB-Consumer-Digital-Index-2018-Report.pdf.
13. Parmanto B, Lewis JAN, Graham KM, Bertolet MH. Development of the Telehealth Usability Questionnaire (TUQ). International Journal of Telerehabilitation. 2016;8(1):3-10.
14. Esper CD, Merola A, Himes L, Patel N, Bezchlibnyk YB, Falconer D, et al. Necessity and feasibility of remote tele-programming of deep brain stimulation systems in Parkinson's disease. Parkinsonism Relat Disord. 2022;96:38-42.
15. Wildenbos GA, Peute L, Jaspers M. Aging barriers influencing mobile health usability for older adults: A literature based framework (MOLD-US). Int J Med Inform. 2018;114:66-75.
16. HotJar.
17. Daniel WW CC. Biostatistics: A Foundation for Analysis in the Health Sciences, 7th Edition. New York: John Wiley & Sons; 2018.
18. Green J, Thorogood N. Qualitative Methods for Health Research: SAGE; 2018 2018/2/26. 440 p.
19. Sauro J, Lewis JR. Quantifying the User Experience: Practical Statistics for User Research: Morgan Kaufmann; 2016 2016/7/12. 350 p.
20. Braun V, Clarke V. Using thematic analysis in psychology. Qual Res Psychol. 2006;3(2):77-101.
21. Dobkin RD, Amondikar N, Kopil C, Caspell-Garcia C, Brown E, Chahine LM, et al. Innovative Recruitment Strategies to Increase Diversity of Participation in Parkinson's Disease Research: The Fox Insight Cohort Experience. J Parkinsons Dis. 2020;10(2):665-75.
22. NIHR.
23. Topol E. The Topol review: preparing the healthcare workforce to deliver the digital future. Health Education England.
24. Silva AG, Caravau H, Martins A, Almeida AMP, Silva T, Ribeiro Ó, et al. Procedures of User-Centered Usability Assessment for Digital Solutions: Scoping Review of Reviews Reporting on Digital Solutions Relevant for Older Adults. JMIR Hum Factors. 2021;8(1):e22774-e.
25. Maggio MG, Luca A, D'Agate C, Italia M, Calabrò RS, Nicoletti A. Feasibility and usability of a non-immersive virtual reality tele-cognitive app in cognitive rehabilitation of patients affected by Parkinson's disease. Psychogeriatrics. 2022;22(6):775-9.
26. Engelsma T, Jaspers MWM, Peute LW. Considerate mHealth design for older adults with Alzheimer's disease and related dementias (ADRD): A scoping review on usability barriers and design suggestions. Int J Med Inform. 2021;152:104494.
27. Martins AI, Silva AG, Pais J, Cruz VT, Rocha NP. The impact of users' cognitive function on evaluator perceptions of usability. Sci Rep. 2022;12(1):13753.
28. Kalia LV, Lang AE. Parkinson's disease. Lancet. 2015;386(9996):896-912.
29. Harte R, Glynn L, Broderick B, Rodriguez-Molinero A, Baker P, McGuiness B, et al. Human Centred Design Considerations for Connected Health Devices for the Older Adult. Journal of Personalized Medicine. 2014;4(2):245-81.

For peer review only

For peer review only - http://bmjopen.bmj.com/site/about/guidelines.xhtml

30. Mele B, Van S, Holroyd-Leduc J, Ismail Z, Pringsheim T, Goodarzi Z. Diagnosis, treatment and management of apathy in Parkinson's disease: a scoping review. BMJ Open. 2020;10(9):e037632.
31. Matthew-Maich N, Harris L, Ploeg J, Markle-Reid M, Valaitis R, Ibrahim S, et al. Designing, Implementing, and Evaluating Mobile Health Technologies for Managing Chronic Conditions in Older Adults: A Scoping Review. JMIR mHealth and uHealth. 2016;4(2):e29.
32. Karagiannidou M, Stevens M, Knapp M, Cyhlarova E. Recruitment into dementia studies: Experiences of researchers using the Join Dementia Research register. Int J Geriatr Psychiatry. 2022;37(1):n/a.

Figure 1: Project Milestones

Figure 2: Website Functionality
[a]Tasks completed by the project Administrator on a separate account
[β]Study screening is performed externally to the website
[γ]Timepoint website user is invited to take part in an interview

For peer review only

For peer review only - http://bmjopen.bmj.com/site/about/guidelines.xhtml

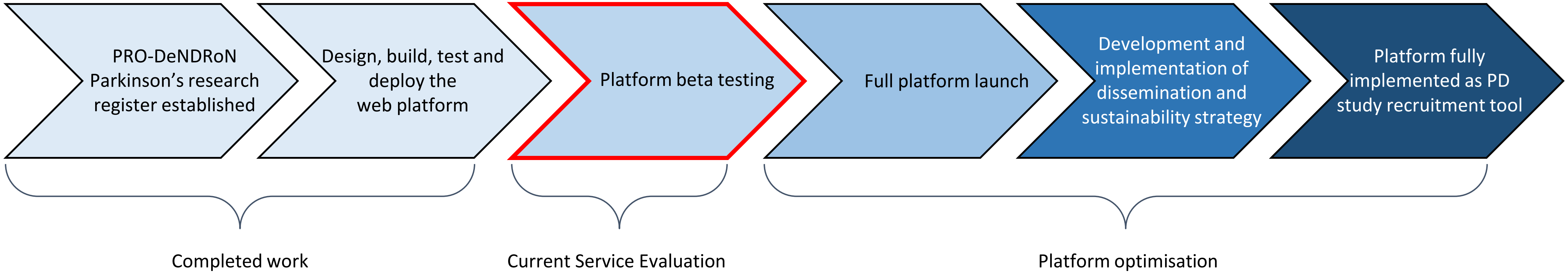
PRO-DeNDRoN Parkinson's research register established
Design, build, test and deploy the web platform
Platform beta testing
Full platform launch
Development and implementation of dissemination and sustainability strategy
Platform fully implemented as PD study recruitment tool
Completed work
Current Service Evaluation
Platform optimisation

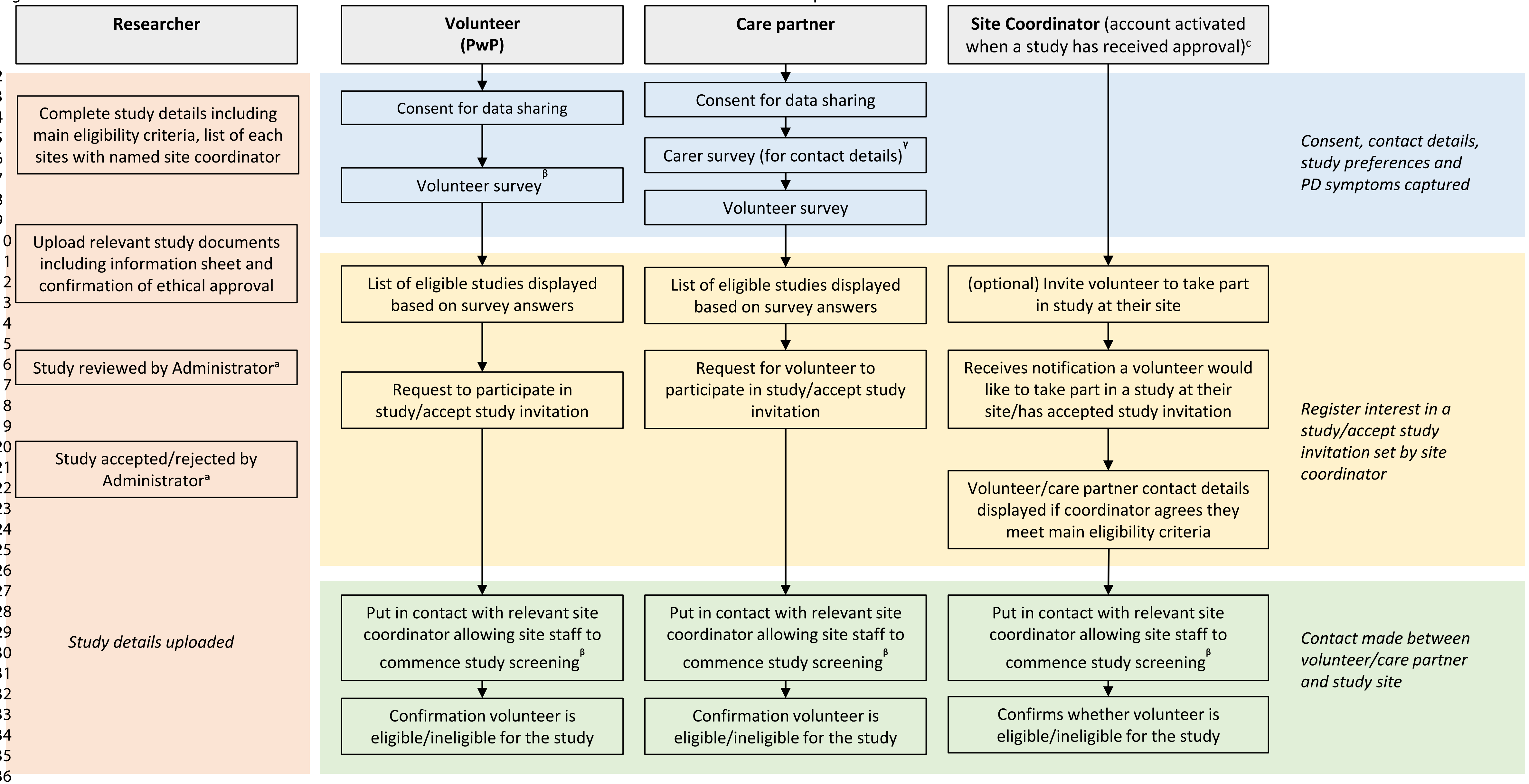
Researcher
Volunteer (PwP)
Care partner
Site Coordinator (account activated when a study has received approval)[c]
Complete study details including main eligibility criteria, list of each sites with named site coordinator
Upload relevant study documents including information sheet and confirmation of ethical approval
Study reviewed by Administrator[a]
Study accepted/rejected by Administrator[a]
Study details uploaded
Consent for data sharing
Volunteer survey[β]
Consent for data sharing
Carer survey (for contact details)[γ]
Volunteer survey
Consent, contact details, study preferences and PD symptoms captured
List of eligible studies displayed based on survey answers
Request to participate in study/accept study invitation
List of eligible studies displayed based on survey answers
Request for volunteer to participate in study/accept study invitation
(optional) Invite volunteer to take part in study at their site
Receives notification a volunteer would like to take part in a study at their site/has accepted study invitation
Volunteer/care partner contact details displayed if coordinator agrees they meet main eligibility criteria
Register interest in a study/accept study invitation set by site coordinator
Put in contact with relevant site coordinator allowing site staff to commence study screening[β]
Confirmation volunteer is eligible/ineligible for the study
Put in contact with relevant site coordinator allowing site staff to commence study screening[β]
Confirmation volunteer is eligible/ineligible for the study
Put in contact with relevant site coordinator allowing site staff to commence study screening[β]
Confirms whether volunteer is eligible/ineligible for the study
Contact made between volunteer/care partner and study site

People with Parkinson's and care partners) Interview Registration Form [to be inserted into JISC survey)

Section 1

The following data is collected so that we are able to contact you to arrange an interview:

First Name:
Surname:
Date of Birth:
Email Address:
Phone Number:

Have you been using the website as a person with Parkinson's or as a care partner?

- Person with Parkinson's
- Care partner

Preferred method of contact:

- Email
- Phone

Section 2

We would like to ensure that the website is as user friendly as possible for everyone. By telling us a little bit more about yourself, you will help us achieve this goal.

Post Code:

Gender
Please select your gender

- Male
- Female
- Other (please specify)

Ethnicity
Please select your ethnicity:

- Asian/Asian British – Indian
- Asian/Asian British – Pakistani
- Asian/Asian British – Bangladeshi
- Asian/Asian British – Chinese
- Asian/Asian British – Any other Asian Background
- Black/African/Caribbean Background – African
- Black/African/Caribbean Background – Caribbean
- Black/African/Caribbean Background – Any other Black/African/Caribbean background
- Mixed Ethnic Group – White and Black Caribbean
- Mixed Ethnic Group – White and Black African

For peer review only

- Mixed Ethnic Group – White and Asian
- Mixed Ethnic Group – Any other mixed ethnic group
- Other Ethnic Group – Arab
- Other Ethnic Group – Berber Arab
- Other Ethnic Group – Ashkenazi Jewish
- White – English/Welsh/Scottish/Northern Irish/British
- White – Irish
- White – Gypsy or Irish Traveller
- White – Any other background

Parkinson's disease diagnosis (not applicable for care partners)

- When were you diagnosed with Parkinson's disease? (please give an approximate date if you are not sure) (dd/mm/yyyy)

Familiarity with digital technology

Please indicate if you are able to do any of the following tasks on a computer, phone or tablet

<u>Managing information</u>

- Use a search engine to look for information online
- Find a website I have visited before
- Download/save a photo I found online

<u>Communicating</u>

- Send a personal message via email or online messaging service
- Carefully make comments and share information online

<u>Transacting</u>

- Buy items or services from a website
- Buy and install apps on a device

<u>Problem solving</u>

- Verify sources of information I found online
- Solve a problem with a device/digital service using online help

<u>Creating</u>

- Complete online application forms which include personal details
- Create something new from existing online images, music or video

For peer review only

Researcher Interview Registration Form (to be inserted into JISC survey)

Section 1

The following data is collected so that we are able to contact you to arrange an interview:

First name:

Surname:

Date of birth:

Phone number:

Email address:

Site name:

Site Post Code:

Study name:

Preferred method of contact:

- Email
- Phone

Section 2

We would like to ensure that the website is as user friendly as possible for everyone. By telling us a little bit more about yourself, you will help us achieve this goal.

Please tell us how you have been using the website

- As a researcher
- As a site coordinator
- As both a researcher and site coordinator

Gender

Please select your gender

- Male
- Female
- Other (please specify)

Ethnicity

Please select your ethnicity:

- Asian/Asian British – Indian
- Asian/Asian British – Pakistani
- Asian/Asian British – Bangladeshi
- Asian/Asian British – Chinese
- Asian/Asian British – Any other Asian Background
- Black/African/Caribbean Background – African
- Black/African/Caribbean Background – Caribbean
- Black/African/Caribbean Background – Any other Black/African/Caribbean background
- Mixed Ethnic Group – White and Black Caribbean
- Mixed Ethnic Group – White and Black African

For peer review only

- Mixed Ethnic Group – White and Asian
- Mixed Ethnic Group – Any other mixed ethnic group
- Other Ethnic Group – Arab
- Other Ethnic Group – Berber Arab
- Other Ethnic Group – Ashkenazi Jewish
- White – English/Welsh/Scottish/Northern Irish/British
- White – Irish
- White – Gypsy or Irish Traveller
- White – Any other background

What type of Trust is your site a part of?
Please select the most relevant Trust type

- Large Acute
- Medium Acute
- Small Acute
- Mental Health
- Other (please specify)
- Not known

How many Parkinson's disease studies have you coordinated?

- Please specify the number of Parkinson's disease studies you have coordinated at your current site, and any previous sites you have worked at. If you are not sure, please give a rough estimate.

Familiarity with digital technology

Please indicate if you are able to do any of the following tasks on a computer, phone or tablet

<u>Managing information</u>

- Use a search engine to look for information online
- Find a website I have visited before
- Download/save a photo I found online

<u>Communicating</u>

- Send a personal message via email or online messaging service
- Carefully make comments and share information online

<u>Transacting</u>

- Buy items or services from a website
- Buy and install apps on a device

For peer review only

Problem solving

- Verify sources of information I found online
- Solve a problem with a device/digital service using online help

Creating

- Complete online application forms which include personal details
- Create something new from existing online images, music or video

For peer review only

Prototype Topic Guide for Interviews with People with Parkinson's and Care Partners

Interviewer introduces themselves

Before we start, is it okay with you if I audio-record this session?

- The reason we record is so we can go back and remember what was said and what wasn't said. Destroyed as soon as study is done, any publications don't use names
    - *Ask again for confirmation once recording is on*
    - If no: "That's fine, is it okay if I type notes as we talk and share those notes with you once we have finished the interview so you can make sure they are accurate?"

The purpose of this study is find out about your experiences of using the website, so we can find out what is working well or what needs to be changed or improved. Your feedback is very valuable for us to evaluate the website.

Today we're going to be going through some questions to discuss your experiences using the website, and then you will have the opportunity to provide feedback on anything else you want to talk about that you feel we haven't covered. If there is anything you don't understand during the interview, feel free to ask!

Before we get into those questions, I just wanted to remind you that all of your answers will be kept confidential and stored on a password-protected computer that only the research team can access. The audio recording that we're taking today will be deleted as soon as it has been transcribed, and any identifying information (like your name) will be removed from the audio recording before it is transcribed.

So before we get started, do you have any questions?

1. General usability

- How would you describe your experience of using the website?
    - *How much did you like or dislike using the website? (Why was this? What made you like/dislike that aspect?)*
    - *How easy or difficult did you find the website to use? (Why was this? What made that aspect easy or difficult to use?)*
    - *What did you think of the colours used? (Why was this? What made you like/dislike the colours?)*

2. Registration and survey completion

- How did you find the process of registering for the website?
    - Do you feel there is anything that could be improved with the registration process?
    - If so, what?
- [PwP only] How did you find the demographic survey?
- [Carer only] How did you find the volunteer survey completion?
    - [Both] Were there any questions that you found difficult?

For peer review only

3. Finding studies

- Did you find it easy to find studies that you might be eligible for?
    - If no, why not?
    - If yes, what was your experience of this?
- What did you think of the study information provided?
    - Is there any other information about a study that you would want to know?
- Did you feel there was anything that could be improved when searching for studies?
    - If so, what?

4. Study enrolment and researcher contact

- Have you enrolled on to any studies through JPR?
    - If yes, what did you find easy about the process?
    - What did you find tricky?
    - Could anything have been clearer?
    - If no, why not?

5. General feedback

- What would you suggest to make the website better?
    - *Can you tell me more about that, and how you think it would improve the website?*
    - *Are there any other suggestion that you think would make the website better?*
- Is there anything else you would like to mention?

For peer review only

Prototype Topic Guide for Interviews with Researchers

Interviewer introduces themselves

Before we start, is it okay with you if I audio-record this session?

- The reason we record is so we can go back and remember what was said and what wasn't said. Destroyed as soon as study is done, any publications don't use names
    - *Ask again for confirmation once recording is on*
    - If no: "That's fine, is it okay if I type notes as we talk and share those notes with you once we have finished the interview so you can make sure they are accurate?"

The purpose of this study is find out about your experiences of using the website, so we can find out what is working well or what needs to be changed or improved. Your feedback is very valuable for us to evaluate the website.

Today we're going to be going through some questions to discuss your experiences using the website, and then you will have the opportunity to provide feedback on anything else you want to talk about that you feel we haven't covered. If there is anything you don't understand during the interview, feel free to ask!

Before we get into those questions, I just wanted to remind you that all of your answers will be kept confidential and stored on a password-protected computer that only the research team can access. The audio recording that we're taking today will be deleted as soon as it has been transcribed, and any identifying information (like your name) will be removed from the audio recording before it is sent to the transcription service.

So before we get started, do you have any questions?

1. **General usability**

- How would you describe your experience of using the website?
    - *How much did you like or dislike using the website?(Why was this? What made you like/dislike that aspect?)*
    - *How easy or difficult did you find the website to use? (Why was this? What made that aspect easy or difficult to use?)*

2. **Registration**

- How did you find the process of registering for the website?
    - Do you feel there is anything that could be improved in terms of being registered as a site study coordinator or researcher for your study? If so, what?

3. **Inviting and enrolling participants**

- Did any volunteers send a request to participate in your study?
    - Have they been enrolled onto the study?
    - If no, why not?
- Did you invite any volunteers to enrol on your study?

For peer review only

- If no, why not?
- Once a volunteer had requested participation, or accepted the study invite, what did you find easy about the enrolment process?
  - What made it tricky?
  - Could anything have been clearer?
  - Did you feel there was anything else that could be improved? If so, what?

4. General feedback

- What would you suggest to make the website better/easier to use/easier to navigate/functions that you would like to see?
  - *Can you tell me more about that, and how you think it would improve the website?*
  - *Are there any other suggestion that you think would make the website better?*
- Is there anything else you would like to mention?

For peer review only
